# A Review of Hand Neuroanatomy, Anesthesia, and Bedside Blockade

**DOI:** 10.7759/cureus.108516

**Published:** 2026-05-08

**Authors:** Aneri U Patel, Alisa O Girard, Alec H Fisher, India M Jones, Matthew M Delancy, Michael J Franco

**Affiliations:** 1 Plastic and Reconstructive Surgery, Cooper Medical School of Rowan University, Camden, USA; 2 Plastic and Reconstructive Surgery, Cooper University Hospital, Camden, USA

**Keywords:** anesthesia, bedside procedure, digital nerve block, hand, neuroanatomy, peripheral nerve block

## Abstract

Many hand-related pathologies can be appropriately managed at the bedside without operative escalation, thereby reducing resource allocation, healthcare costs, and exposure to general anesthesia. Plastic surgery trainees have variable experience and knowledge in managing hand pathologies outside the operating room. A comprehensive clinical reference for hand anesthesia has yet to be seen in the current plastic surgery literature. This review aims to guide the safe and effective administration of regional anesthesia in the hand and wrist for bedside procedures. This review was guided by clinical experience at a high-volume Level I trauma center in the Northeastern United States. A board-certified hand surgeon and plastic surgery residents discussed practical skills and concepts commonly applied to hand procedures performed in the emergency department. A review of the literature was performed, investigating anesthetic agents, considerations for use, neuroanatomy of the hand, and versatile blockade techniques. In contrast to local infiltration, nerve blockade reaches deeply innervated structures while avoiding multiple painful injection sites, tissue distortion, and wound contamination. Nerve blocks are discouraged in patients who are unable to communicate symptoms of intraneural injection. However, bedside nerve blocks in awake children achieve high efficacy and low complication rates, particularly when ultrasound-guided injection or sedation is employed. Local anesthetic systemic toxicity can be avoided through knowledge of anatomical landmarks, anesthetic characteristics, and appropriate dosing. The hand has complicated yet predictable neurovascular anatomy. With proper anesthetic administration, a remarkable degree of patient comfort and a high level of procedural complexity can be achieved outside the operating room.

## Introduction and background

Hand-related pathologies represent a growing proportion of emergency department (ED) encounters in the United States [1]. Lacerations of the wrist, hand, or digit account for more than 9 million ED visits annually [2]. Many presenting hand complaints can be appropriately managed at the bedside, including abscess drainage, foreign body removal, laceration repair, dislocation/fracture reductions, and digit revision amputation, without further hospital admission or operative escalation [3]. Definitive management in the ED therefore reduces resource allocation, general anesthesia exposure, healthcare costs, and duration of patient stay [3]. Local infiltration anesthesia and peripheral nerve blocks have made the treatment of common hand conditions possible with minimal patient distress [4].

Plastic surgery residents are routinely tasked with managing traumatic hand injuries and consultations. However, trainees have variable experience with hand pathology, and exposure is often limited relative to other topics [5]. The current literature offers recommendations to advance resident competency in hand conditions, including adjunctive hand curricula and simulation-based training [6]. However, a comprehensive instructional reference for bedside hand anesthesia has yet to be seen. The purpose of this review is to guide the plastic surgery resident in the safe and effective administration of regional anesthesia in the hand and wrist.

## Review

Methods

A board-certified hand surgeon (MJF) and residents in plastic surgery (AOG, AHF, IMJ, MMD) discussed the challenges of common bedside hand procedures at a Level I trauma center and agreed on important topics for resident education: (1) neuroanatomy of the hand and wrist, (2) anesthetic agents, and (3) appropriate techniques for regional anesthetic administration. A review of the literature was subsequently performed without restriction on publication dates. Topical keywords (e.g., digital nerve block, radial nerve block, regional anesthesia of the hand) were used. Prospective and retrospective studies, case studies, reviews, technique papers, and textbooks were included in this review.

Results

Local Anesthetics

It is critical that the plastic surgery resident be well-versed in the action of different local anesthetics and their safe maximum dosages. Local anesthetics inhibit voltage-gated sodium channels in neurons, which interrupts membrane conduction and prevents nociceptive signaling to the central nervous system [7]. Amino amides (e.g., lidocaine, mepivacaine, bupivacaine, ropivacaine) undergo enzymatic degradation in the liver and are subsequently excreted in the urine. Amino esters (e.g., procaine, tetracaine, chloroprocaine) are hydrolyzed by plasma pseudocholinesterase into the metabolite para-aminobenzoic acid (PABA), which has a propensity to trigger hypersensitivity reactions, albeit rarely reported [8].

Beyond traditional formulations, novel delivery systems have been developed to modify pharmacokinetics and increase the duration of action of local anesthetics. Liposomal bupivacaine is a sustained-release formulation created to prolong analgesia and reduce postoperative opioid use. However, evidence of superiority over conventional bupivacaine remains mixed among recent randomized controlled trials [9,10]. In addition to pharmacologic modifications, catheter-based peripheral nerve block (CPNB) techniques are alternative strategies for prolonging anesthesia after surgery that can be titrated to the desired effect in the perioperative period [11,12]. Long-acting local anesthetics are primarily used as infusates for CPNB, with exclusive basal infusions, repeated bolus doses, or a combination of the two [12]. Studies have shown that the use of levobupivacaine via CPNB has been associated with effective postoperative analgesia, demonstrating a decline in postoperative opioid use [12,13].

The most common complications of local anesthetic use include infection, nerve injury, vascular injury, hematoma, and inadvertent injection into vascular or nervous structures [14]. Local anesthetic systemic toxicity (LAST) is a dose-dependent spectrum of sequelae that results from excessive tissue administration or direct intravascular delivery. Blood levels of lidocaine typically reach a peak 6-12 hours after tissue infiltration [14,15]; however, this can be altered by the location of injection, pKa of the anesthetic, acidity of the tissue, and presence of vasoconstrictive medications [16]. Symptoms of LAST can be observed when serum concentrations approach 5 mcg/mL [14]. The earliest symptoms include muscle twitching, tinnitus, headaches, confusion, and a metallic taste. At serum concentrations of 10 mcg/mL and higher, patients can suffer from loss of consciousness, convulsions, coma, respiratory depression, and death [14-16]. When toxicity is suspected, it is critical to stop agent injection, call for help, stabilize vital signs, and treat seizures (e.g., with benzodiazepines) [17]. Prompt administration of 20% intravenous lipid emulsion therapy should be initiated as definitive treatment to sequester circulating anesthetics. An initial bolus of 100 mL over 2-3 minutes should be followed by an infusion of 200-250 mL over 15-20 minutes [18]. For patients weighing less than 70 kg, an initial bolus of 1.5 mL/kg should be followed by a 0.25 mL/kg/min infusion. If the patient remains unstable, repeat bolus or double infusion, with a maximum lipid dose of 12 mL/kg [18].

Despite prior dogmas about epinephrine use in the hand and fingers, a substantial body of modern literature supports its safety in distal extremity and digit use, with reviews demonstrating no cases of digital necrosis when used at standard concentrations [19-22]. Epinephrine exerts local vasoconstrictive effects, thereby limiting blood loss and ensuring good visibility of the field of injury. Vasoconstriction also limits the diffusion of anesthetics to surrounding tissues, which enables a longer anesthetic effect and may increase the allowable dose of local anesthetics [21]. Additional clinical benefits include a reduced need for a tourniquet, thereby mitigating complications associated with prolonged tourniquet use. While caution is warranted in patients with compromised circulation, epinephrine can be safely used in otherwise healthy patients [23]. As such, appropriate dosing varies among anesthetic formulations (Table 1) [18,24-33].

**Table 1:** Maximum safe doses of commonly used local anesthetic agents in adults *Indicates the two most frequently used agents at the authors’ institution.

Type	Generic Name	Brand Names (®)	Adult Maximum Dosing (mg/kg)				Onset of Action (Minutes)	Duration of Effect (Minutes)	
			Without Epinephrine		With Epinephrine			Without Epinephrine	With Epinephrine
			(mg/kg)	(mg)	(mg/kg)	(mg)			
Ester	Procaine [24,25,28,30]	Novocain	10	350-1000	14	600	5	15-30	30-90
	Tetracaine [25,28,30]	Pontocaine	2	N/A	2	N/A	7	120-240	240-480
	Chloroprocaine [18,28,30]	Nesacaine	11	800	14	1000	6-12	30-60	70-90
Amide	Lidocaine* [24,25,28-30]	Xylocaine	3-5	300-350	7	500	<1	30-120	60-400
	Prilocaine [26,28-30]	Citanest	6-7	400	8-10	600	5-6	30-120	60-400
	Mepivicaine [24-30]	Carbocaine, Polocaine	5	300-400	7	500-550	3-20	30-120	60-400
	Bupivacaine* [24-26,28-30]	Marcaine, Sensorcaine	2-2.5	175	3	225	2-8	120-240	240-420
	Ropivacaine [26,27,30]	Naropin	2.9-3	200-225	3-4	250	1-15	120-240	300-480
	Levobupivacaine [29,31-33]	Chirocaine	2	200	3	225	6-15	522-960	510-750

Anesthetic concentration refers to grams of anesthetic per 100 mL. Therefore, a 1% concentration of anesthetic is equivalent to 1 g/100 mL or 10 mg/mL. The anesthetic concentration, maximum safe dose, and patient weight are needed to calculate the maximum volume of anesthetic that may be safely infiltrated (Equations 1 and 2) [34,35].

Equation 1. Calculation of the maximum safe dose of local anesthetic infiltration, full equation.

\( \text{Max volume (ml)} = \frac{ \text{Max safe dose } \left( \frac{\text{mg}}{\text{kg}}\right) \times \text{ Weight (kg)} }{ \text{Concentration } \left( \frac{\text{g}}{100\,\text{ml}}\right) \times \frac{1000\,\text{mg}}{1\,\text{g}} } \)

Equation 2. Calculation of the maximum safe dose of local anesthetic infiltration, simplified equation.

\( \text{Max volume (ml)} = \text{Max safe dose }\left(\frac{\text{mg}}{\text{kg}}\right) \times \text{Weight (kg)} \div 10 \div \text{Concentration (%)} \)

Nerve Anatomy and Blockade

**Figure 1:**
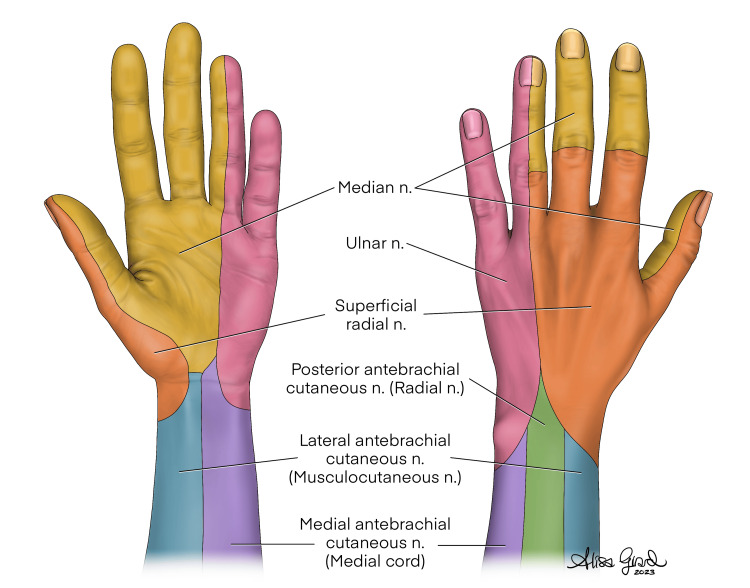
Dermatomes of the hand and distal antebrachium The image is an original illustration created by Alisa O. Girard, MD, using Procreate (Savage Interactive Pty., Hobart, Australia).

**Figure 2:**
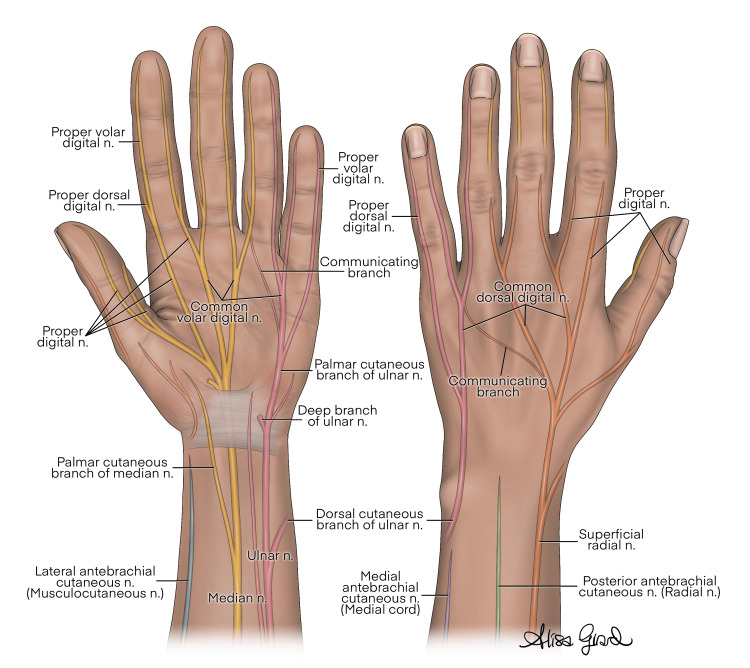
Nerves and branches supplying the hand and distal antebrachium The image is an original illustration created by Alisa O. Girard, MD, using Procreate (Savage Interactive Pty., Hobart, Australia).

**Figure 3:**
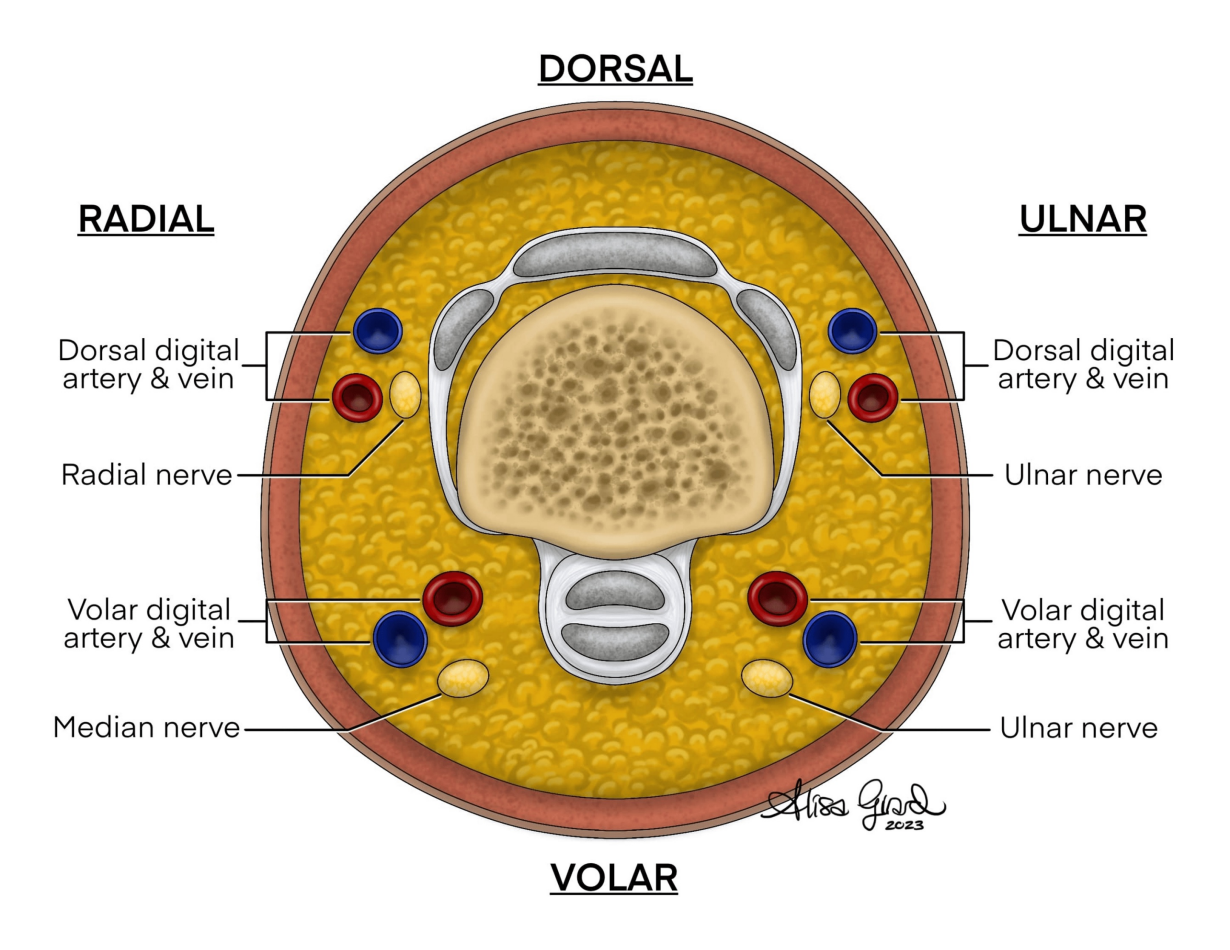
Neurovascular bundles in a cross-section of the proximal left ring finger The image is an original illustration created by Alisa O. Girard, MD, using Procreate (Savage Interactive Pty., Hobart, Australia).

Sensory innervation of the hand is supplied by the median, ulnar, and radial nerves (Figure 1 and Figure 2) [13]. The relatively consistent course of nerves and nearby landmarks enables reliable blockade of peripheral nerves and large regions of the hand. Given the distribution of multiple nerves to each digit, digital blocks may instead be recommended for pathology limited to specific digits (Figure 3) [36].

Median Nerve Anatomy

The median nerve is derived from the lateral and medial cords of the brachial plexus (C5-T1). In the arm, it first courses laterally to the brachial artery between the biceps brachii and brachialis muscle bellies. At the level of the coracobrachialis insertion, the median nerve crosses and then travels medial to the brachial artery [37]. At the cubital fossa, the median nerve gives off an articular sensory branch to the elbow joint and a motor branch to the two heads of the pronator teres muscle as it passes between them. In the antebrachium, the nerve courses between flexor digitorum profundus (FDP) and flexor digitorum superficialis (FDS) muscles, where it gives off its two main branches: the anterior interosseous nerve (motor) in the proximal antebrachium and the palmar cutaneous branch of the median nerve (PCBm) distally [37,38]. The PCBm originates 4.1-8.4 cm proximal to the distal volar wrist crease, traversing superficial to the flexor retinaculum into the hand, where it supplies sensation to the radiovolar palm [13].

The median nerve continues distally beneath the transverse carpal ligament. As it exits the carpal tunnel, the median nerve immediately gives off a recurrent motor branch to the thenar eminence and first two lumbricals and then splits into a smaller radial and larger ulnar division [38]. The radial division gives rise to three proper digital nerves supplying the radial thumb, ulnar thumb, and radial index finger. The larger ulnar branch divides into two common digital nerves that travel to the level of the second and third webspace, just distal to the metacarpal heads. Each common digital nerve gives rise to two proper digital nerves, which further divide into volar and dorsal branches supplying the distal digit [39].

Ulnar Nerve Anatomy

The ulnar nerve is the terminal branch of the medial cord (C8, T1, and variably C7) [40]. In the axilla, the ulnar nerve passes anterior to the insertion of the teres major and long head of the triceps and medial to the brachial artery. It then courses posteromedially before piercing the medial intermuscular septum 8 cm proximal to the medial epicondyle. It further descends through a band of deep brachial fascia, the Arcade of Struthers, as it passes anterior to the medial head of the triceps muscle [40,41]. The cubital tunnel is defined by a fibrous retinaculum, “Osborne’s ligament,” the medial epicondyle, olecranon process, and the joint capsule with its medial collateral ligament. Within the cubital tunnel, just distal to the medial epicondyle, the ulnar nerve gives off multiple motor branches to supply flexor carpi ulnaris (FCU) and the ulnar half of FDP [42].

After emerging deep to Osborne's ligament, the ulnar nerve traverses between FCU muscle heads, emerging on the volar surface of FDP. Approximately halfway along the antebrachium, the palmar cutaneous branch arises from the ulnar nerve proper [40,41]. A dorsal cutaneous branch originates 5 cm proximal to the ulnar styloid and emerges dorsally to cross the wrist. Here, the dorsal branch divides into digital branches to supply the ulnodorsal small and radiodorsal ring fingers [41].

The ulnar nerve proper, traveling with the ulnar artery, becomes superficial before entering the hand through Guyon’s canal. This canal is a fibro-osseous tunnel between the pisiform and the hook of hamate and is roofed by the superficial volar carpal ligament [40,41]. Within this canal, the ulnar nerve bifurcates into superficial (mixed) and deep (motor only) branches. The superficial branch innervates the palmaris brevis muscle and sensation over the hypothenar muscles. It then divides into the fourth common digital nerve and the ulnar proper digital nerve to the small finger [41].

Radial Nerve Anatomy

The radial nerve is a terminal branch of the posterior cord (C5-T1). In the brachium, the radial nerve runs posterior and inferior to the axillary artery, passing anterior to the insertion of the latissimus dorsi and between the medial and long heads of the triceps to the posterior surface of the humerus. It then courses in the spiral groove of the humerus and emerges superficially within 2 cm of the lateral epicondyle [43]. The radial nerve gives off a posterior cutaneous branch just proximal to the lateral epicondyle, which provides sensation to the elbow and dorsal antebrachium. At the antecubital fossa, the nerve pierces the lateral intermuscular septum and enters the anterior compartment of the antebrachium between the brachialis and the brachioradialis. Here, it divides into two terminal branches: a superficial sensory branch (SRN) and a deep motor branch. SRN emerges dorsally from beneath the brachioradialis tendon 8-9 cm proximal to the radial styloid and continues toward the hand in a subcutaneous plane [44,45]. The SRN divides 5 cm proximal to the radial styloid into volar and dorsal branches bearing variable branching patterns and courses. The dorsal branch supplies the dorsoradial aspect of the hand, including the dorsal thumb, index finger, middle finger, and radial half of the ring finger. The volar branch contributes sensation to the radial palm [44].

Digital Nerve Anatomy

Apart from the thumb, each digit contains two volar and two dorsal neurovascular bundles. Proper digital arteries arise from common digital arteries distal to the analogous branching pattern of digital nerves. As each proper digital nerve passes the interdigital webspace, it transitions from a dorsal to a volar position relative to the associated proper digital artery and vein [46]. Each proper digital nerve contributes small branches along the length of the digit [47]. It is important to note that variations in the classically described branching pattern and nerve distributions are quite common; it could be stated that variation is the rule, rather than the exception [48]. For example, there is notable variation in the contribution of volar versus dorsal digital nerves to the sensation of the dorsal distal region of the digits [49]. Anomalous interconnections have also been reported between sensory nerve branches, resulting in preserved distal sensation contrary to proximal blockade of the classically supplying nerve [50]. A Berrettini anastomosis, present in most patients, involves the donation of an ulnar common digital nerve branch to the median-derived common digital nerve at the webspace between the middle and ring fingers [51]. Several other anastomoses between the median and ulnar nerves have been observed more proximally with varied sensory and motor involvement [50]. With that said, digital anesthesia may be best approached via transthecal or transmetacarpal blocks rather than more proximal peripheral nerve blocks.

Median Nerve: Block at the Wrist

**Figure 4:**
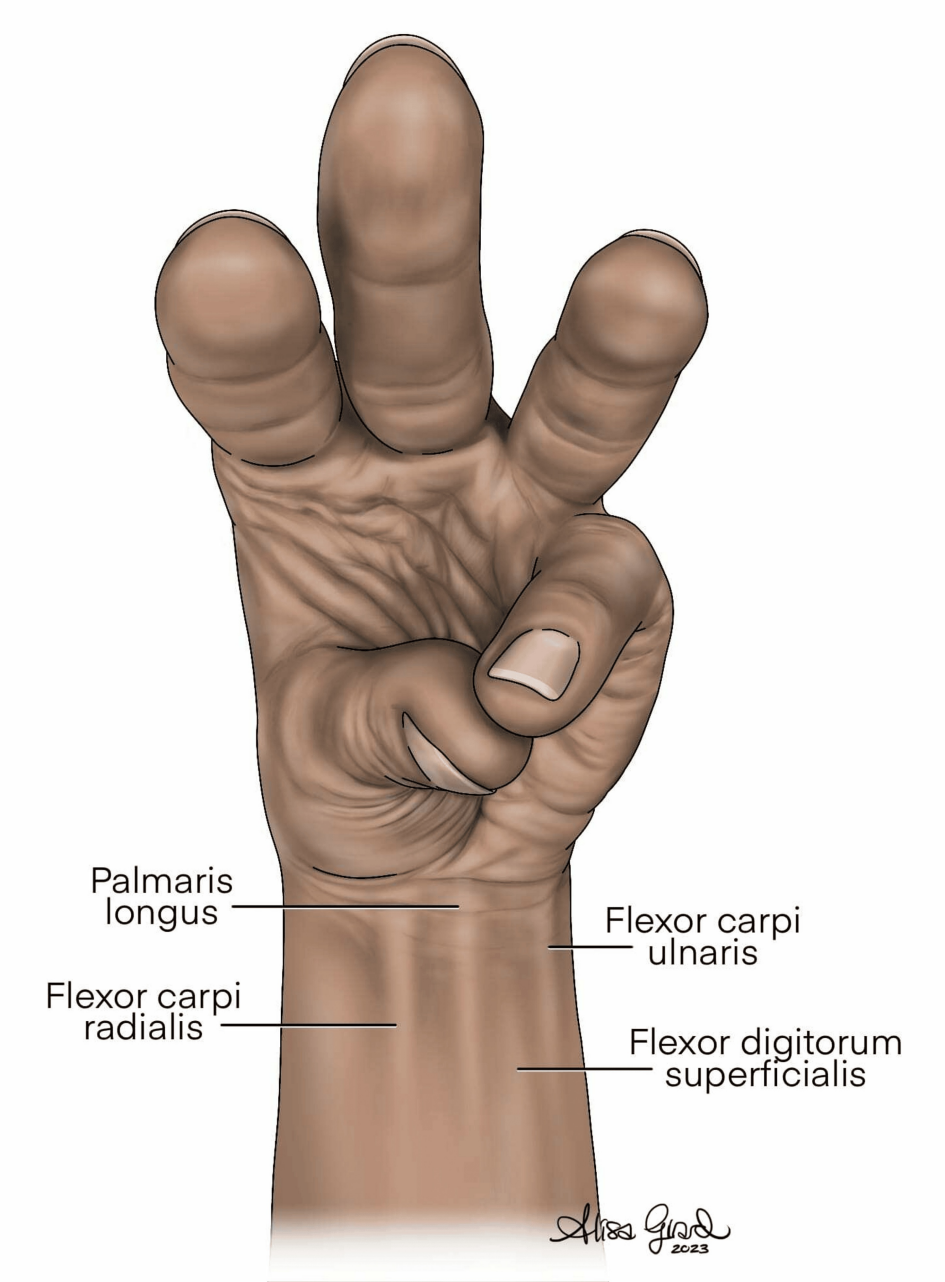
Identification of the palmaris longus tendon via hand and wrist posturing The image is an original illustration created by Alisa O. Girard, MD, using Procreate (Savage Interactive Pty., Hobart, Australia).

To block the median nerve at the wrist, the needle is inserted between the palmaris longus and flexor carpi radialis tendons, at the level of the proximal wrist crease. One can identify the palmaris longus by asking the patient to oppose the thumb to the small finger while flexing the wrist (Figure 4). Absence of a palmaris longus tendon is common, in which case the needle is inserted on the ulnar side of the flexor carpi radialis tendon [52]. The needle should enter the skin at a 90° angle and be advanced through the flexor retinaculum, sometimes with a palpable "pop" [52]. Beneath the retinaculum, 3-5 cc of local anesthetic should be injected. When withdrawing the needle, 1 cc of local anesthetic should be injected above the flexor retinaculum to block the superficial palmar branch [53].

Median Nerve: Ultrasound-Guided Approach

Peripheral nerve blocks of the hand are commonly performed in the ED using traditional anatomic landmarks. Though safe and effective, other useful applications such as ultrasonography have been demonstrated to reduce rescue anesthesia and/or analgesia when performing nerve blocks [54]. Studies in the current literature discuss ultrasound-guided median nerve block in the antecubital fossa [55-57]. Very few have commented on its use for the blockade of the median nerve at the wrist, which may be explained by the anatomic complexity of this region, including the numerous tendons that surround the median nerve, making nerve identification more challenging [55,58].

On ultrasound, the median nerve appears as a hyperechoic structure with flexor tendons directly below it. This is best visualized with the transducer placed over the wrist crease. The needle should be advanced distally toward the transducer at a 45° angle [59]. Consistent anesthesia achieved with smaller volumes and fewer complications via ultrasound-guided proximal nerve blocks suggests a promising role for ultrasound-guided median nerve block at the wrist [55].

Ulnar Nerve: Block at the Wrist

**Figure 5:**
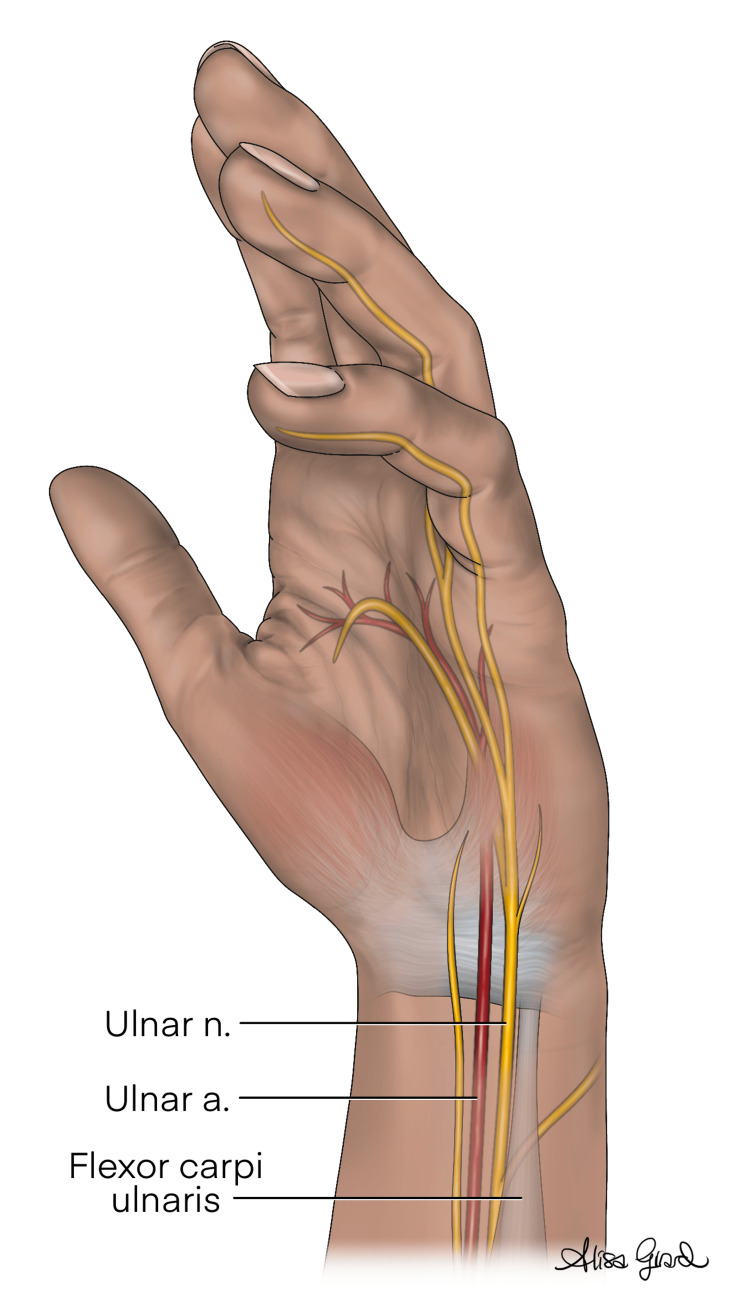
Proximity of the ulnar nerve to the flexor carpi ulnaris tendon at the wrist The image is an original illustration created by Alisa O. Girard, MD, using Procreate (Savage Interactive Pty., Hobart, Australia).

At the wrist, the ulnar artery lies just radial to the ulnar nerve [53]. There are three widely used approaches for ulnar nerve block at the wrist: the volar approach, the ulnar approach, and the transtendinous volar (TTV) approach. In all three techniques, the needle is inserted perpendicular to the long axis of the arm, with the hand supinated on a flat surface [60]. The approaches differ in their insertion points. The more popular volar approach targets the area between the FCU and the distal ulna. For this approach, the FCU is first palpated at its insertion site at the pisiform. The needle is then inserted at the proximal wrist crease, parallel along the radial border of FCU [61]. For the ulnar approach, the needle is inserted parallel to the proximal wrist crease and posterior to the FCU tendon sheath (Figure 5). Needle insertion just ulnar to the FCU tendon with a radially directed needle decreases the likelihood of injecting into or damaging the artery [61]. This approach may also block the dorsal cutaneous branch of the ulnar nerve, thus preventing an additional needle stick [52].

For the TTV approach, the needle is inserted through the midportion of the FCU tendon sheath until there is loss of resistance, which indicates that the needle has traversed the tendon. A bolus of anesthetic is then delivered. Ultimately, the three approaches differ in the distance between the needle tip and the ulnar nerve or ulnar artery, as demonstrated by cadaveric studies [61]. The injection distance from both the artery and nerve is greatest for the ulnar approach, followed by TTV and then volar. The findings of the cadaveric studies help explain the relatively high (up to 50%) intra-arterial puncture rate for the volar approach, as compared to the 0% intra-arterial puncture rate for the ulnar approach, suggesting that the more popular approach [60,61].

Radial Nerve: Block at the Mid-antebrachium

The radial nerve has a relatively superficial course through the upper extremity, making it easily accessible without an ultrasound or nerve stimulator [62]. As such, the superficial radial nerve (SRN) can be blocked with good reliability before it branches. At a point 8 cm proximal to the radial styloid, the nerve is radial to the extensor carpi radialis brevis and ulnar to the brachioradialis [44]. One must be mindful to avoid the cephalic vein, which is readily visible at this point. In this interval, inject 5-7 mL of local anesthetic in a subcutaneous plane [63]. The authors prefer to deposit a large subcutaneous wheal in this location and then gently massage the anesthetic along the course of the nerve.

Radial Nerve: Block at Anatomical Snuff Box

**Figure 6:**
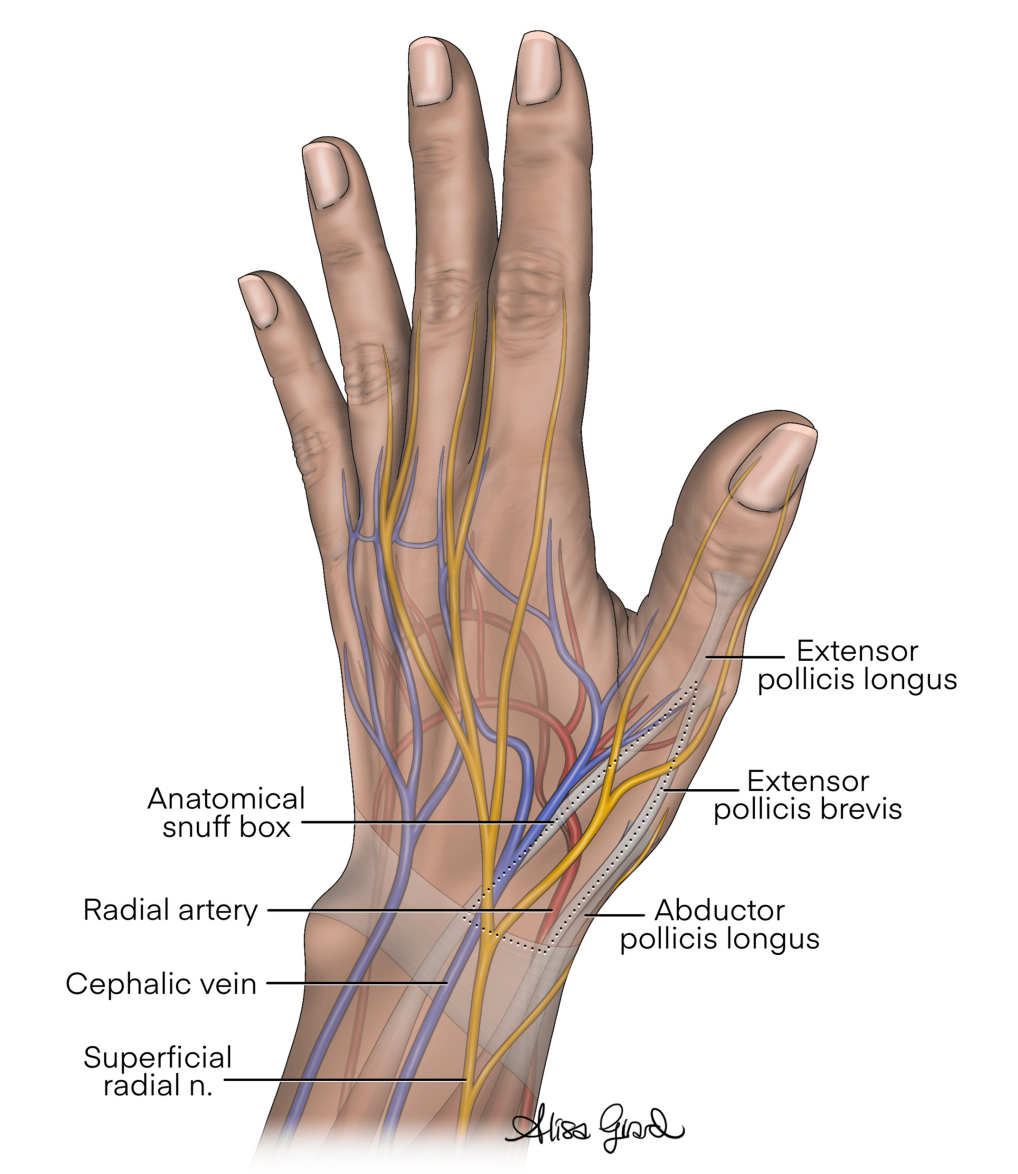
Radial nerve course through the anatomical snuff box The image is an original illustration created by Alisa O. Girard, MD, using Procreate (Savage Interactive Pty., Hobart, Australia).

The anatomic snuff box is a triangular depression of the dorsal hand that is situated just distal to the extensor retinaculum, bounded radially by abductor pollicis longus and extensor pollicis brevis and ulnarly by extensor pollicis longus [64]. The SRN courses through the anatomic snuff box within 2 mm radial to the radial artery before it gives off arterial branches to the thumb and palmar arch (Figure 6) [45]. The nerve can safely be anesthetized without risk of arterial injury at this level by adhering to two principles. First, palpate the radial artery in the anatomic snuff box and insert the needle just radial to the area of palpation. Second, keep the needle superficial, advancing the tip no deeper than the subcuticular level of the tendinous boundaries of the snuff box. Then, inject 3-7 mL of local anesthetic in a subcutaneous plane, assuring negative aspiration with no radiating pain prior to injection [65].

Digital Nerves: Dorsal Webspace Block

Digital nerve blocks may be performed by three commonly described techniques: the dorsal webspace block, the transthecal block, and the volar/dorsal transmetacarpal block. Each technique differs in its point of injection and potential associated risks. The dorsal webspace block is historically known as the traditional approach for achieving digital nerve anesthesia [66]. The patient’s hand should be pronated and rested on a flat surface [67]. Holding the syringe perpendicular to the length of the digit, the needle should then be gently inserted into the webspace 1 cm distal to the metacarpophalangeal joint (MCPJ), where the anesthetic should be gradually administered. The needle is advanced toward the volar aspect of the webspace while infiltrating the surrounding tissues with up to 3 mL of anesthetic, being careful not to pierce the volar aspect of the webspace [68,69].

Digital Nerves: Transthecal Block

Unlike the traditional dorsal webspace block, which threatens the digital neurovascular bundles either directly or indirectly via elevated compartment pressure, the transthecal digital block has gained popularity for its technical ease and reduced neurovascular risk profile [70]. This block utilizes the flexor tendon sheath for anesthetic distribution. The patient’s hand should be supinated on a flat surface. At the level of the A1 pulley (the level of the palmar digital crease or metacarpal head is equally effective), a 24-gauge needle is advanced toward the digital midline into the flexor tendon sheath until the needle tip touches bone. The needle is withdrawn 2-3 mm and redirected 45° along the digit axis. Approximately 2-3 mL of anesthetic can then be infiltrated into the flexor sheath with minimal resistance. With the non-injecting hand, apply pressure proximally to facilitate distal dispersion of the anesthetic within the tendon sheath [13,70].

**Figure 7:**
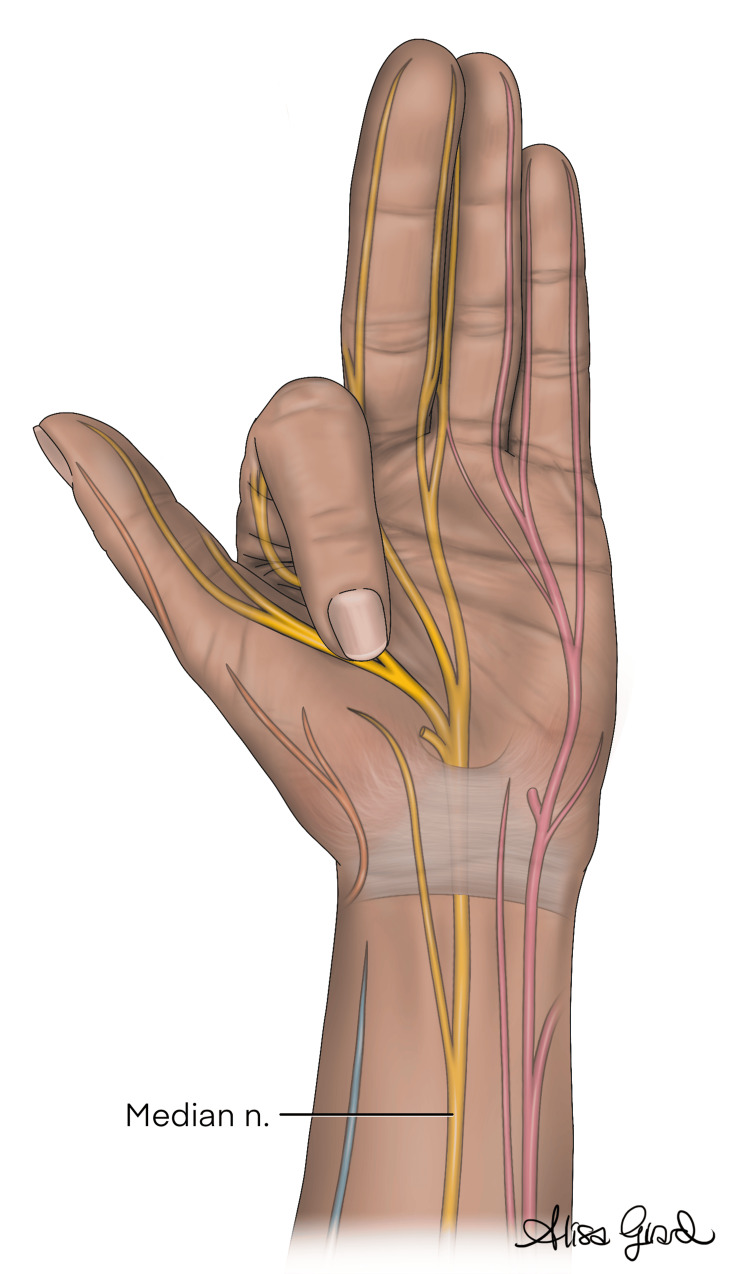
Localization of median nerve digital block at the volar thenar eminence The image is an original illustration created by Alisa O. Girard, MD, using Procreate (Savage Interactive Pty., Hobart, Australia).

The thumb can be more challenging to adequately anesthetize due to its increased neurotization from the SRN dorsally, possible contributions from the lateral antebrachial cutaneous nerve radiodorsally, and the median nerve volarly, which commonly bifurcates in the thenar eminence and then again at the level of the metacarpophalangeal joint [71,72]. Use of a single volar injection carries the risk of inadequate dorsal anesthesia. To block the thumb, one can use a volar transthecal block in conjunction with a 1-2 mL dorsal subcutaneous skin wheal to anesthetize the dorsal sensory nerves [73]. Another effective technique for volar anesthesia is to deliver an anesthetic bolus into the volar thenar eminence, bathing the median-derived digital nerve prior to its division. This area can be identified by having the patient flex the index finger at the MCPJ and proximal interphalangeal joint (PIPJ) such that the index volar pad contacts the thenar eminence (Figure 7). The area of contact approximates the site of digital nerve bifurcation and is a relatively safe deep injection space [71].

Digital Nerves: Transmetacarpal Block

The transmetacarpal block can be performed from a dorsal or volar approach. With the pronated hand placed flat on the table, a 24-gauge needle is inserted approximately 1 cm proximal to the MCPJ on the dorsal aspect of the hand. The needle is advanced at a 90° angle until the deep surface of the volar skin is reached, typically felt when resistance is met and slight tenting of the volar skin is noted. Approximately 3-5 mL of anesthetic is injected slowly as the needle is withdrawn dorsally. Prior to completely removing the needle, a subcutaneous dorsal wheal will block the dorsal sensory branches of the radially or ulnarly oriented nerves. This technique requires two injections, one on each side of the metacarpal head [74].

Comparison of Digital Nerve Block Techniques

The transthecal block has gained popularity for its technical ease as a single-injection approach and safety when compared to the traditional dorsal webspace block, which threatens digital neurovascular bundles via elevated compartment pressures [75]. Yet, the transthecal and volar transmetacarpal approaches come with their own risk of injury and infection to both the tendon sheath and the tendons themselves [68]. A meta-analysis study has shown that while volar approaches are associated with greater pain reported by patients, most probably due to increased innervation and thicker glabrous skin, a single-injection approach is still preferred by patients [52,68]. Moreover, there seems to be no significant difference in the overall efficacy of anesthesia among the three approaches, including time to onset and duration of anesthesia [68]. When choosing an ideal digital nerve block technique, the site of surgery may also be considered.

Discussion

The hand has a complex yet predictable nervous anatomy, wherein structural variations have been well elucidated. Regional anesthesia can therefore be achieved with high reliability and safety in the awake patient. There exists a broad selection of anesthetic techniques that can be utilized either independently or in combination. However, the decision to perform a peripheral nerve block over local infiltration depends on an array of pathological, anatomical, and patient-specific factors.

Peripheral Nerve Block Versus Local Infiltration

Whether anesthesia of the hand is better achieved via local tissue infiltration or peripheral nerve block is a matter of debate and personal preference. For digital anesthesia, patients report less pain with nerve blocks [76]. Benefits of local infiltration include local hemostatic effects at wound edges (with epinephrine), rapid onset, and ease of use for those unfamiliar with hand neuroanatomy. Disadvantages include pain and risk to local structures with multiple needle passes, greater anesthetic volumes required for larger wounds, distortion of tissue architecture, wound contamination, increased pain with injection into an area with high nociceptive activity or increased extracellular acidity (i.e., infection), and poor blockade of deeply innervated structures (i.e., bone and muscle) [76,77]. Apart from small lacerations, the authors typically prefer nerve blocks. However, it is recommended that nerve blocks not be used in a patient who is unable to communicate symptoms of intraneural injection (e.g., intubated, sedated). Real-time feedback with the patient should be elicited such that if the patient experiences an electric-shock sensation or paresthesia in the nerve distribution, the needle should be withdrawn immediately. Nerve blocks may also be inappropriate in patients with injuries that distort critical anatomic landmarks [78].

Considerations in the Pediatric Population

Nerve blocks are particularly challenging in the pediatric population due to heightened distress, poor compliance with instructions, sudden movements during the procedure, and limited capacity to communicate symptoms of intraneural injection [79]. Subsequently, general anesthesia is commonly applied before nerve blockade in children [80]. Despite this, nerve blocks in awake children have been documented with high efficacy and low complication rates when performed by experienced hands [81]. Ultrasound-guided injection is generally encouraged [82], as traditional anatomic landmark-based approaches in adults are often poorly adapted to the pediatric patient [83]. Depending on the patient’s age, anticipatory discussion, parent instruction, and patient distraction can facilitate injection and the procedure [84]. When poor tolerance is suspected, the patient may receive minimal-to-moderate sedation with continuous monitoring [85]. In the authors’ experience, awake sedation enables safe and efficient evaluation and procedure at the bedside.

## Conclusions

With proper administration of local anesthetic, a remarkable degree of patient comfort and a high level of procedural complexity can be achieved in the awake patient. A foundational understanding of local anesthesia, peripheral nerve anatomy, and common block techniques is encouraged to ensure appropriate bedside management of hand pathologies commonly encountered by the plastic surgery resident. This review provides a detailed overview of these topics to engender confidence and competence in hand training.
